# A mixed‐methods exploration of non‐attendance at diabetes appointments using peer researchers

**DOI:** 10.1111/hex.12959

**Published:** 2019-09-05

**Authors:** Claire Eades, Helen Alexander

**Affiliations:** ^1^ Faculty of Health Sciences and Sport University of Stirling Stirling UK; ^2^ NHS Lanarkshire Bothwell UK

**Keywords:** diabetes, health psychology, non‐attendance, patient and public involvement

## Abstract

**Background:**

Non‐attendance at diabetes appointments is costly to the health service and linked with poorer patient outcomes.

**Objective:**

Peer researchers aimed to conduct interviews and survey people who miss appointments about their beliefs and perceptions regarding their diabetes and diabetes appointments.

**Design:**

A mixed‐methods cross‐sectional design with interviews conducted by peer researchers with diabetes and a questionnaire was used.

**Setting and participants:**

Peer researchers conducted semi‐structured telephone interviews in one health board in Scotland with ten people who had missed diabetes appointments. A further 34 people who had missed appointments completed a questionnaire. The study was informed by two psychological theories (the Theory of Planned Behaviour and the Self‐Regulation Model), and interviews were analysed using thematic analysis.

**Results:**

Interviewees planned to attend appointments but practical barriers, low perceived value of appointments and the feeling that diabetes had little impact upon their lives’ emerged as key reasons for missing appointments. Questionnaire data supported these findings and showed that respondents perceived diabetes to have only mildly serious consequence and cause limited concern and emotional impact. Participants’ understanding of their condition and perceptions of personal control and treatment control were low. Gender, perceived behavioural control and emotional representations were significantly associated with the number of appointments missed in the previous year.

**Conclusions:**

These findings highlight the importance of psychological variables in predicting non‐attendance at diabetes appointments and provide avenues for how non‐attendance might be tackled.

## 1 BACKGROUND

Many people who have diabetes do not regularly attend their diabetes‐related appointments. Estimates of the prevalence of missed diabetes vary but two large studies in the United States reported that 16.2% of people with diabetes missed their last primary care appointment^1^ and 12% of people missed more than 30% of schedules primary care appointments in 1 year.^2^ Non‐attendance at scheduled appointments has consequences both for the health service and for the person with diabetes. Non‐attendance increases the cost of delivering care, reduces available appointments and increases waiting times for other patients.^3^ People with diabetes who do not attend clinic appointments tend to have poorer glycaemic control, more complications, more frequent hospital admissions and increased all‐cause mortality.^1^, ^2^, ^3^, ^4^, ^5^ Previous research on the topic of non‐attendance at diabetes appointments has generally focused on demographic and clinical factors such as age and gender. If non‐attendance is to be tackled, it is important to seek the opinions of people with diabetes themselves and to investigate psychosocial factors such as beliefs and attitudes that are potentially amenable to change.^6^

Patients have historically played a passive role in health research, but the benefits of a more active role are increasingly being recognized and patient involvement in health‐care research is recommended in health‐care policy in the UK.^7^ Patients can be involved in a range of activities from the design of research to conducting research and presenting the findings, and there is evidence to suggest that patient involvement can help to improve the credibility and relevance of research.^8^, ^9^ A systematic review of the impact of patient involvement in research noted that there were benefits at all stages of the research process including increased enrolment rates and development of research questions, questionnaires and interview schedules that were more user focused.^9^ To our knowledge, there has been no previous research investigating non‐attendance at diabetes appointments that has involved patients who have diabetes in the research process. Training people with diabetes to survey peers who have failed to attend appointments could generate novel and valuable findings because of the shared experience of those carrying out the work and those being surveyed.

Two psychological models of illness/health behaviour have been widely used in research on long‐term conditions generally, and specifically to understand non‐attendance in diabetes appointments^6^: the Theory of Planned Behaviour (TPB^10^) and the Self‐Regulation Model of Illness Behaviour (SRM^11^). The TPB states that voluntary behaviours are largely predicted by our intentions regarding the behaviour. Intentions in turn are determined by our attitude towards the behaviour (our judgement of whether the behaviour is a good thing to do), subjective norms (our judgement of what important others think of the behaviour) and perceived behavioural control (PBC; our expectation of how successful we will be in carrying out the behaviour^10^).

The SRM proposes that people interpret information about a potential illness to create a ‘lay’ view or representation of the illness. The coping responses employed by an individual, for example, adhering to treatment regimens and attending appointments, are said to be related to the illness representations they hold and to their appraisal of how successful they perceive the chosen coping responses to be. Illness representations are proposed to be formed around six different themes: identity (label or diagnosis of illness), cause (factors believed to have caused the illness), timeline (expected duration of illness), consequences (expected effects of illness on physical, social and psychological wellbeing), control/cure (extent to which illness can be controlled/cured) and illness coherence (how well the person understands their illness^11^).

Both of these models have been previously been applied to understand non‐attendance at diabetes appointments and could therefore provide a potentially valuable framework for a peer‐led investigation of this issue.^6^ The SRM and TPB offer different approaches to understanding attendance at diabetes clinic appointments with the SRM focusing on patients’ beliefs about diabetes, whereas the TPB is concerned with beliefs about the actual act of attending appointments. As there is no clear evidence to suggest which approach might be most appropriate, both models were used to inform the present study. The aim of the study was to conduct peer‐led interviews and surveys with people who miss diabetes appointments to assess their beliefs and perceptions about their diabetes and attendance at diabetes appointments.

## 2 METHODS

### 2.1 Study design

This study used a mixed‐methods cross‐sectional design to explore non‐attendance at diabetes appointments in one health board in Scotland. People with diabetes (peer researchers) were involved in the conception and design of the study and were trained to carry out semi‐structured telephone interviews in the first phase of the study. The findings of these interviews informed the content of a questionnaire in the second phase of the study. It was originally intended that the first author would carry out telephone interviews to provide a comparator to the peer researcher interviews but a very low response rate meant this was not possible. Ethical approval was obtained for this study from the West of Scotland NHS Research Ethics Committee 4 (reference number: 13/WS/0177). Participants in the telephone interviews were sent information sheets and provided informed consent verbally. Information sheets were sent out with the questionnaires, and completion of the questionnaire was taken to imply consent. The authors elect to not share data.

### 2.2 Peer researcher training

Four people with diabetes who were interested in being peer researchers were identified from an existing diabetes patient group and trained to conduct semi‐structured telephone interviews but only three of peer researchers were available at the time of data collection to conduct interviews. Two of the peer researchers were female, one was aged between 50 and 60, two were aged over 60, two peer researchers had type 1 diabetes, and one had type 2 diabetes. Training for peer researchers was developed and delivered by the first author, who at the time of the study was a Registered Health Psychologist and PhD candidate. The training was based on training delivered by colleagues of the first author for a previous research project using peer researchers and on resources from the INVOLVE website (INVOLVE is a national advisory group that supports greater public involvement in health and social care research). Training included content on confidentiality, ethics, interviewing techniques, reflexivity and dealing with distressed participants and provided opportunities for role play to give peer researchers practical experience of these topics (See Table 1 for a full outline of topics covered in training). In total, two evenings and one full day of training were provided, and peer researchers were informed they could request additional training at any point as required, either individually or as a group. Both authors independently assessed the peer researchers’ interview skills during role play, and all four met the required standard for the agreed criteria. The first author telephoned peer interviewers before and after they carried out their first interview to check they were comfortable with the interview process and to reflect on their experience of conducting it. Contact after the first interview was made according to the preferences of the peer researcher.

**Table 1:** Outline of peer researcher training content

	Content overview	Specific content
Evening 1	Introduction to research	Introduction to research Introduction to this research project Public involvement in research
	Research ethics	Introduction Ethical Consent Confidentiality and Anonymity Safety of interviewer and interviewee
	Qualitative research questions	Types of questions Why questions should be open, neutral and singular.
Evening 2	Interviewing skills	Planning the interview Starting the interview Listening Prompting and probing Finishing the interview Potential pitfalls
Day Session	Interview rehearsal	Opportunity to go over any areas again Development of own interview schedule for practice. Full rehearsal of an interview with practice schedule. Full rehearsal of interview using the interview schedule for this study.
	Close of training and feedback	Distribution of interview materials Feedback will be sought on learner's perceived confidence and knowledge to conduct interviews. What happens next—outline contact details and planned support.

### 2.3 Participants and recruitment

People with diabetes who had previously failed to attend diabetes clinic appointments were recruited from a single health board in Scotland, UK. English‐speaking patients, aged over 18, with type 1 or type 2 diabetes, were eligible for inclusion if they had missed three or more diabetes clinic appointments in the previous 24 months (either in hospital or at their general practice). Twenty General Practices and two secondary care diabetes clinics agreed to identify patients for this study, although not all practices claimed the small remuneration offered to cover the administrative cost of this, so fewer than 20 may have managed to distribute the information. An initial contact letter was drafted for the study and sent out by a member of the existing clinical care team, along with an information sheet about the study. Patients selected to take part in the telephone interviews were asked to return a slip to indicate that they were interested and agreeing to their details being passed on to the research team. Patients selected to take part in the questionnaire part of the study were sent a paper copy of the questionnaire along with an initial contact letter. Different participants took part in the telephone interviews and questionnaire.

### 2.4 Data collection

#### 2.4.1 Telephone interviews

Telephone interviews were conducted by peer researchers between December 2014 and February 2015. They used an interview guide informed by underlying theory and their own experience. The semi‐structured format of the interviews ensured that the topics of interest were covered whilst allowing interviewees the freedom to discuss any issues not included in the guide. The main topics were as follows: experiences of having diabetes, treatment and control of diabetes, emotional impact of diabetes, understanding of diabetes, pros and cons of attending diabetes appointments, and barriers and facilitators to attending diabetes appointments. Table 2 gives example questions for each topic and shows which constructs from the theoretical framework that these questions relate to. Interviews lasted between 8 and 27 minutes and were audio‐recorded with the participant's permission and transcribed verbatim by a professional transcription service. Peer researchers were offered, but chose not to accept any remuneration for their involvement in the study.

**Table 2:** Summary of interview topic guide and the theoretical constructs from which each topic is derived

Topic area	Guide questions	Construct/theory
Experiences of diabetes	Can you tell me about your diabetes?	Identity and Timeline/SRM
	How much do you feel your diabetes affects your life?	Consequences/SRM
Treatment and control of diabetes	How much control do you feel you have over your diabetes?	Personal control/SRM
	How much do you think your treatment can help your diabetes?	Treatment control/SRM
	How much do you think attending diabetes clinic appointments can help your diabetes?	Treatment control/SRM Attitudes/TPB
Emotional impact of diabetes	How much does your illness affect you emotionally (eg does it make you angry, scared, upset or depressed)?	Emotional representations/SRM
	How concerned are you about your diabetes?	Emotional representations/SRM
Understanding of diabetes	How well do you feel you understand your illness?	Coherence/SRM
Barriers and facilitators to attending diabetes appointments	Can you tell me a bit about how you feel about attending appointments to do with your diabetes?	Attitude/TPB
	Can you tell me about anything you like/don't like about attending diabetes appointments?	Attitude/TPB
	Are there any people who you think would approve/disapprove of you attending diabetes appointments?	Social norms/TPB
	Can you tell me about anything that makes it easier/harder for you to attend diabetes appointments?	Perceived Behavioural Control/TPB
	If you did want to attend a clinic appointment, how sure are you that you would be able to?	Perceived Behavioural Control/TPB

Only a few of the practices involved reported how many people they contacted with information about taking part in a telephone interview, but extrapolating from those who did, we estimate that 200 received this request. A total of 14 people completed a return slip to indicate they were willing to be contacted. The peer researchers were able to make contact and interview 10 of the 14 potential participants but problems with recording meant that verbatim transcripts were only used in the analysis for 7 interviews and field notes for the remaining 3. Table 3 lists the participants in the telephone interviews and their characteristics.

**Table 3:** List of participants quoted in the text and characteristics

Participant number	Characteristics	Data type
P1	Type 1, male, diagnosed 25 y ago	Verbatim transcript
P2	Type 2, male, diagnosed 9 y ago	Verbatim transcript
P3	Type 2, female, diagnosed 6 y ago	Verbatim transcript
P4	Type 2, female, diagnosed 13 y ago	Verbatim transcript
P5	Type 2, female, diagnosed 9 y ago	Verbatim transcript
P6	Type 2, male, diagnosed 18 y ago	Verbatim transcript
P7	Type 2, female, diagnosed 4 y ago	Verbatim transcript
P8	Type 2, unknown duration	Field notes
P9	Type 1, unknown duration	Field notes
P10	Type 2, unknown duration	Field notes

#### 2.4.2 Questionnaire

Questionnaires were sent to patients from June to September 2016 and again from June to November 2017 to boost the response rate. The questionnaire collected data on participants’ age, gender, type of diabetes and the number of diabetes appointments missed in the last two years. It also included questions assessing the components of the TPB, the SRM. Components of the SRM were assessed using the Brief Illness Perceptions Questionnaire (Brief IPQ) which is a standardized and validated measure for assessing illness perceptions.^12^ Items on the IPQ are scored on a scale of 1 to 10 with one item assessing each of the dimensions of illness perceptions (consequences, timeline, personal control, treatment control, identity, coherence, emotional representation and concern). An overall score for illness perceptions was calculated by adding together responses to the items as outlined by Broadbent et al.^12^ A higher overall illness perception score indicates a more threatening view of diabetes as an illness. Responses to the item assessing perceived cause of diabetes were grouped according to categories such as hereditary and lifestyle. Cronbach's alpha for the IPQ items was 0.75.

No standardized measure currently exists for assessing the components of the TPB. The guide produced by Francis et al^13^ was used to develop an appropriate TPB questionnaire for the present study. This guide recommends conducting an ‘elicitation study’ to inform development of the TPB measure. An elicitation study is a qualitative investigation that aims to establish the most salient beliefs about a particular behaviour in the population in question. In this study, the telephone interviews were used to form an elicitation study to identify attitudes and barriers to attendance at diabetes clinic appointments. The most salient attitudes and barriers identified in the interviews were developed into questions to be included in the questionnaire. The format of the TPB questions meant that reliability analysis was not appropriate.^13^ Full details of how TPB components were measured in the questionnaire are outlined in Table 4.

**Table 4:** Measurement of TPC constructs in questionnaire

Construct	Items	Scale	Scoring
Attitude			
Behavioural beliefs	1. Going to diabetes clinics will cause me to worry about my condition (unlikely/likely). 2. Going to diabetes clinics will help me to manage my condition (unlikely/likely).	1 to 7	Item scores were multiplied as follows: 1*3, 2*4. Overall attitude score was the sum of the resulting scores. Overall attitude scores had a range of −42 to +42 with a negative score representing a negative attitude and a positive score a positive attitude to attending appointments.
Outcome evaluation	3. Worrying about my condition is extremely undesirable/extremely desirable. 4. Managing my condition is extremely undesirable/extremely desirable.	−3 to +3	
Subjective norm			
Injunctive norms	1. My family/friends think that I should/I should not attend diabetes clinic appointments. 2. My doctors think that I should/I should not attend diabetes clinic appointments.	−3 to +3	Item scores were multiplied as follows: 1*4, 2*5, 3*6. The overall subjective norm score was calculated by taking summing the three resulting scores. Overall subjective norm scores had a range of −63 to +63 with a negative score representing negative social pressure and a positive score positive social pressure towards attending appointments.
Descriptive norm	3. Other people with diabetes do/do not attend all of their clinic appointments.	−3 to +3	
Motivation to comply	4. What my family/friends think I should do matters to me (not at all/very much). 5. What my doctor thinks I should do matters to me (not at all/very much). 6. Doing what other people with diabetes do is important to me (not at all/very much).	1‐7	
Perceived behavioural control			
Control beliefs	1. It will be difficult to get transport to my diabetes clinic appointments (strongly disagree/strongly agree). 2. Diabetes clinic appointments are likely to be at a time of day that doesn't suit me (strongly disagree/strongly agree) 3. It will be difficult for me to remember to attend my appointment (strongly disagree/strongly agree).	1‐7	Item scores were multiplied as follows: 1*4, 2*5, 3*6. Overall perceived behavioural control score was the sum of the resulting scores. Overall perceived behavioural control scores had a possible range of −63 to +63 with a negative score representing low perceived behavioural control and a positive score high perceived behavioural control for attending appointments.
Perceived power	4. When it is difficult to get transport to my diabetes clinic appointment I am less likely/more likely to attend. 5. When clinic appointments are at a time of day that doesn't suit me I am less likely/more likely to attend. 6. When clinic appointments are at a time of day that doesn't suit me I am less likely/more likely to attend.	−3 to +3	
Intention	1. I intend to attend all of my diabetes clinic appointments in the next year (strongly disagree/strongly agree).	1‐7	A score of one indicated low intention and a score of seven indicated high intention to attend diabetes appointments

### 2.5 Data analysis

A thematic analysis of the interview data was conducted independently by the first author.^14^ Thematic analysis involves the identification of themes in interview transcripts. This is achieved through reading and re‐reading the data to become familiar with the content. Regular recurring experiences and feelings described by participants are manually identified and then formed into themes which give an overall view of the way that participants feel about the service. This method was chosen as it organizes and minimizes data whilst retaining detail. Although no independent analysis of the data was carried out, a draft of the themes was shared and discussed with the peer researchers to check the interpretations made by the first author.

Data collected from the questionnaire were analysed using SPSS for windows version 25. The characteristics of the sample, and TPB and SRM components were analysed using descriptive statistics. Comparisons of SRM and TPB components across demographic groups were carried out using Mann‐Whitney tests. This non‐parametric alternative to the independent t test was appropriate with the small sample size and violations of normality found in the data. Mann‐Whitney tests were carried out for each demographic variable (gender, age and type of diabetes) with the demographic variable entered as the independent variable and the SRM and TPB components entered as the dependent variables. Poisson regression analysis was conducted to assess whether TPB and SRM components and demographic variables predicted self‐reported attendance at diabetes clinic appointments.

## 3 RESULTS

### 3.1 Telephone interviews

Three reasons for non‐attendance at diabetes appointments emerged from the interviews conducted by peer researchers: two of which were explicitly stated by participants and a third was implicit in the interview data. The two explicitly stated reasons for non‐attendance were practical barriers to attending and low perceived value of attending. A perception of diabetes as having a limited impact on participants’ lives emerged as a third implicit reason for not attending diabetes appointments. The findings regarding these reasons for non‐attendance at diabetes appointments are discussed in detail below with supporting verbatim quotes from participants identified by participant number. Table 5 summarizes the main themes and subthemes from the telephone interviews.

**Table 5:** Main themes and subthemes from qualitative telephone interviews

Theme	Subthemes
Practical barriers to attendance	Competing demands
	Transport
	Forgetting
Value of appointments	Positive perceptions of value
	Provoking worry/fear
	Lack of value
Perceived impact of diabetes	Lack of impact on day to day life
	Type 2 controlled by medication

#### 3.1.1 Practical barriers

Many participants mentioned that competing demands for their time, such as work or family commitments, made it difficult for them to attend appointments.

We’ve got quite a small team, and, um, and over the last couple of years I’ve been made the assistant manager, so I’ve got quite a lot of responsibilities…and that, so it is…it is harder to get away for appointments. (P1)

I don’t have a problem attending them. It’s just that the way things have turned out this month… Well, I should have…this month, between the end…the end of November and the beginning of December, it should have been, but I’ve been up and down, up and down to the hospital, in fact I’m not long in from the hospital. (P2)

Participants made it clear that they did not intend to miss appointments but that these competing demands, such as participant two's hospital visits to an ill relative, took precedence. Some participants suggested that a wider range of appointment times and having fewer, longer appointments that addressed various aspects of their care would make it easier for them to attend.

There’s maybe so many appointments…within the space of…within the space of like maybe two or three months. I don’t…sometimes I think there’s too many. There’s too many appointments at the one time. (P1)

For most participants, travelling to appointments was not a barrier to attending but some participants did experience difficulties particularly with those appointments that were further away from home. Participant five talked about problems with her mobility and the difficulties she had in getting public transport to hospital appointments.

If I want my eyes checked, I have to go to Hamilton. Well, my daughter used to live close and she used to take me to Hamilton. But now she’s not here anymore, I can’t get there. So I rang them up and cancelled the appointment. (P5)

Two participants stated that the main reason they had missed appointments was because they forgot. They both commented that the length of time that often passed between appointments made them difficult to remember.

Oh, I’m up for it and then I forget and that’s as simple as that. I just forget. I just…I go, right, I’m definitely going and then the next time I go oh god I forgot it again. I don’t know exactly when it is. (P3)

Sometimes I forget my appointments. Sometimes you get them in…it’s maybe a year… between them you know…and you…you’ve lost the card and you say, oh when do I go again? And you forget all about them. (P4)

#### 3.1.2 Value of appointments

The majority of participants had something positive to say about attending appointments, often in relation to their practice nurse. Some participants felt that they learned something from appointments and that staff gave them the time they needed.

Oh they’re very good. She’s…she’ll…she talks to me…she doesn’t just, er, do what she has to do and chase me out. She listens to me. (P5)

However, participants also mentioned that there were aspects of appointments that put them off attending. Participant one discussed how he often felt fearful before an appointment, worrying that they might find something wrong.

It’s always quite daunting when you’re going to…when you’re going to a clinic in case they tell you something you’re not going to be happy with. (P1)

Given the worries he had about his health participant, one did not perceive it as helpful when staff stressed the potential consequences of his condition or reprimanded him, although he recognized that they did this for his benefit.

I don’t like getting told off when I’ve no’ been kind of…took my meds. (P1)

Sometimes they can scare you a bit when they’re… When they’re telling you things, not saying like…obviously all the problems that comes out of…problems with your eyes, your kidneys, they’ve told you people can lose limbs and all that, it’s…it’s something that scares you. (P1)

One participant with type 2 diabetes was quite frustrated as she felt she got very little from attending appointments about her diabetes and did not feel she was listened to.

But the appointments at the hospital are just a waste of time. A total waste of time. I’d…I went once, the first couple of times I went you’re there practically all afternoon. Erm, you…you know, they take bloods and then you go in and see the consultant…who pretty much just stays, stay on what you’re on. And I’m saying to him, I can’t understand it…because my bloods are so up and down, why can’t I test my bloods? (P7)

#### 3.1.3 Perceived impact of diabetes

Underlying participants’ discussion about the reasons for non‐attendance at appointments was a sense that their condition was not something that concerned them, and many participants had little to say about their condition which was reflected in the short length of some interviews. Although some participants stated that they did worry about their diabetes and mentioned complications that they had experienced, the majority of participants felt that it had very little effect on their day to day lives.

No. I don’t really take it as an illness, to be quite honest with you…just carry on, you know what I mean. It’s just like you never had it, know what I mean, kind of thing. (P4)

There was a perception among participants with type 2 diabetes that their condition was controlled by the medication they took, rather than by self‐management behaviours. This belief seemed to further reduce concern about their condition.

The tablets just…they…they take it all away and as long as I…that…that’s it. There’s nothing really to it. (P3)

A number of participants showed very little understanding of their condition, and one could not say which type of diabetes she had.

I can’t tell you, er, what type I’ve got. It’s the one that’s…that’s, erm, controlled by tablets. (P3)

### 3.2 Questionnaire

Of the 405 questionnaires distributed, 35 completed ones were returned, although one was excluded from the analysis as the participant reported their age as being younger than 18. The majority of respondents were male (n = 22) and had type 1 diabetes (n = 21; one participant did not know what type of diabetes they had). The mean age of respondents was 49 years old (range 19‐84). Table 6 shows mean scores for TPB and SRM components for the whole sample and the results of one‐way ANOVAs comparing theory components according to gender, type of diabetes and age (younger = 18‐54 and older = 55‐84). The reported number of missed appointments ranged from 0 to 8 with a mean of 2.7 (SD 2.5).

**Table 6:** Results of descriptive statistics Mann‐Whitney comparing TPB and SRM components according to gender, type of diabetes and age

Theory component (possible range of scores)	Overall Median (IQR) n = 34	Median men (IQR) n = 22	Median women (IQR) n = 12	U	*P* (Exact Sig.)	Median type 1 (IQR) n = 21	Median type 2 (IQR) n = 12	U	*P* (Exact Sig.)	Median young (IQR) n = 20	Median old (IQR) n = 14	U	*P* (Exact Sig.)
Theory of planned behaviour													
Attitude (−42‐+42)	7.0 (13.2)	9.0 (20.0)	3.0 (17.0)	79	.155	0.0 (18.3)	7.0 (16.0)	85.5	.252	9.5 (19.3)	4.0 (16.0)	102	.501
Subjective Norm (−63‐+63)	24.3 (28.8)	33 (37.5)	31.5 (23.8)	103	.792	34.5 (28.3)	24.0 (45.0)	74.5	.110	34.5 (24.0)	22.0 (39.5)	62.5	.049
Perceived behavioural control (−63‐+63)	−11.8 (19.2)	−11 (24.5)	−26 (36.5)	106.5	.765	−13.5 (25.8)	−12.0 (29.0)	102	.819	−20.0 (22.8)	−9.0 (36.0)	73	.082
Intention (1‐7)	6.0 (1.8)	7.0 (2)	7.0 (3)	118	.782	7.0 (4.0)	7.0 (1.0)	118	.954	7.0 (3.0)	7.0 (2.0)	131	.957
Self‐regulation model													
Consequences (1‐10)	6.3(3.0)	7.0 (7.0)	6.0 (4.0)	122.5	.897	7.0 (8.0)	6.0 (3.0)	99	.431	6.0 (7.0)	6.0 (3.0)	122.5	.785
Timeline (1‐10)	9.6 (1.1)	10.0 (0)	10.0 (3)	93	.228	10.0 (0)	10.0 (2.0)	89	.239	10.0 (0)	10.0 (1.0)	123	.813
Personal control (1‐10)	4.9 (2.6)	4.0 (4)	5.0 (3)	79.5	.058	4.0 (7.0)	5.0 (2.0)	118	.782	4.0 (5.0)	5.0 (3.0)	121.5	.522
Treatment control (1‐10)	2.2 (2.1)	1.0 (2.0)	2.0 (3.0)	119	.811	1.5 (3.0)	1.0 (2.0)	112	.774	1.0 (2.0)	1.0 (2.0)	120.5	.730
Identity (1‐10)	6.2 (2.7)	7.0 (8.0)	6.3 (3)	120	.683	7.0 (4.0)	5.0 (5.0)	91.5	.200	7.0 (4.0)	6.0 (6.0)	81.5	.039
Concern (1‐10)	6.5 (3.1)	7.0 (5)	6.0 (5.0)	112	.618	7.0 (6.0)	5.0 (4.0)	86	.254	7.0 (5.0)	6.0 (4.0)	122.5	.785
Coherence (1‐10)	4.0 (2.7)	2.0 (3.0)	5.5 (3.0)	58	.010	2.0 (4.0)	5.0 (5.0)	68	.044	3.0 (5.0)	5.0 (5.0)	103	.334
Emotional representation (1‐10)	6.3 (3.5)	7.0 (7.0)	8.0 (3.0)	99.5	.245	8.0 (7.0)	7.0 (5.0)	125.5	.985	8.0 (9.0)	7.0 (6.0)	122.5	.616
Overall IPQ score (8‐80)	46 (13.1)	46.0 (21.5)	52 (13.0)	82.5	.146	48.5 (24.5)	48.0 (15.0)	92	.476	47.5 (26.0)	48.0 (15.0)	114.5	.833

Mean scores for the TPB components across the whole sample outlined in Table 6 show that participants held weakly positive attitudes and perceived there to be moderately positive social pressure to attend diabetes appointments. Although intentions to attend appointments in the next year were high, perceived behavioural control was low. Mean overall illness perception scores indicated a perception of diabetes as neutral/weakly threatening. The mean scores for the individual dimensions of illness perceptions indicate that participants perceived diabetes to have only mildly serious consequences on their lives and cause mild concern and emotional impact. Participants’ perceptions of how much personal control they had over their condition were low, and perceptions of how helpful treatment was for controlling their diabetes were lower still. Mean coherence scores show that participants felt they had a poor understanding of their condition, and mean timeline scores indicate that they viewed their condition as being long‐lasting. The most common causes of their diabetes reported by participants with type 1 diabetes were genes, poor diet and immune system. The most commonly stated causes of type 2 diabetes were being overweight, poor diet, lack of exercise and stress/depression.

The results of the Mann‐Whitney tests comparing TPB and SRM theory components by gender age and type of diabetes showed significant differences in some theory components (see Table 6 for full results). There were significant differences in coherence between men and women with men having lower median scores for both of these constructs meaning they felt they had less control over, and a poorer understanding of their condition. Significant differences were also found in coherence by type of diabetes with people with type 1 diabetes having lower median scores. Significant differences were found in subjective norm, perceived behavioural control and identity between younger and older participants. Younger participants had higher median scores for subjective norm suggesting they felt stronger social pressure to attend appointments but lower median perceived control suggesting that they felt less able to attend appointments. Median identity scores were higher for younger participants meaning that they experienced more symptoms than older participants.

Poisson regression was carried out to predict the number of missed appointments based on gender, type of diabetes, age, TPB components and SRM components. The coefficients, confidence intervals and significance levels for the variables entered in the Poisson regression can be found in Table 7. The overall regression model was significant (*P* < .01). Individual variables that were significantly associated with an increased risk of missing appointments included female gender, higher emotional representation score, higher attitude score and lower perceived behavioural control.

**Table 7:** Poisson regression for number of missed appointments

	Exp (B)	95% CI	*P*
Demographic variables			
Male	0.47	0.22‐0.97	.040
Female	1.0		
Type 1 diabetes	0.88	0.15‐5.29	.890
Type 2 diabetes	1.0		
Age	0.99	0.96‐1.03	.865
Theory of planned behaviour components			
Attitude	1.05	1.01‐1.09	.010
Subjective norm	0.99	0.97‐1.02	.660
Perceived behavioural control	0.93	0.89‐0.97	.001
Intention	0.72	0.48‐1.07	.101
Self‐regulation model components			
Consequences	0.71	0.47‐1.06	.097
Timeline	1.68	0.88‐3.20	.118
Personal control	1.27	0.85‐1.90	.244
Treatment control	0.81	0.49‐1.34	.413
Identity	1.11	0.84‐1.48	.458
Concern	1.16	0.90‐1.50	.246
Coherence	0.83	0.63‐1.10	.190
Emotional representation	1.32	1.03‐1.69	.031

## 4 DISCUSSION

This study is the first, to our knowledge, to have people with diabetes conduct research to explore reasons for non‐attendance at diabetes appointments and builds upon the small body of work exploring the influence of psychological variables on non‐attendance. Understanding factors that influence attendance at diabetes appointments that are potentially amenable to change is vital if non‐attendance is to be tackled.

The interviews conducted by peer researchers revealed that although participants generally planned to attend their appointments, practical barriers to attending, low perceived value of appointments and the feeling that diabetes had little impact upon their lives emerged as key reasons for missing appointments. The data collected by the peer researchers accorded well with the data collected in the questionnaire phase of the study where respondents also reported that they intended to attend appointments but perceived themselves to have limited control and did not hold particularly positive attitudes towards attending their appointments. The questionnaire data provided further understanding of the perception highlighted in the interviews that diabetes had little impact on participants’ lives. Questionnaire respondents viewed diabetes as a long‐lasting condition but perceived it to have only mildly serious consequences and cause limited concern and emotional impact. Participants’ understanding of their condition and perceptions of personal control and treatment control were all low in the present study. Together, these findings highlight a number of practical barriers, beliefs and perceptions underlying non‐attendance at clinic appointments that need to be addressed. Significant differences were found by gender, age and type of diabetes in some beliefs and perceptions suggesting that any intervention addressing these beliefs and perceptions may need to be tailored to the individual.

There is only limited previous research using psychological theory to investigate non‐attendance at appointments. Lawson et al^15^ also investigated attendance at diabetes appointments using the SRM and reported that people with type 1 diabetes who did not seek regular care had low perceptions personal control, more serious perceptions of consequences and longer timeline than those people who did attend appointments. The findings of the present study were largely consistent with the Lawson et al^15^ study with the exception of the finding regarding the serious consequences of their condition.

A qualitative study by Lawson et al^16^ exploring non‐attendance in nine people with type 1 diabetes reported that participants could be divided into three group: the high fear group, patient as expert group and low arousal/motivation group. The high fear group experienced anxiety about long‐term complications of diabetes which was heightened by attending appointments; the patient as expert group felt they had control over their diabetes; and the low arousal/low motivation group showed an absence of strong emotion or concern towards their condition and did not view it as a serious condition. Although there was one participant in the present study who reported experiencing fear about attending appointment and receiving bad news about their condition and complications, the majority of participants in our study fitted in to the patient as expert and low arousal groups described by Lawson et al.^16^

Some of the differences in illness perceptions between the present study and the Lawson et al studies^15^, ^16^ may be explained by the fact that we only included people who had missed appointments, whereas Lawson et al^15^, ^16^ compared non‐attenders with people who did attend appointments. We also included people with type 1 and type 2 diabetes, whereas Lawson et al^15^, ^16^ only included people with type 1 diabetes. Although type 1 and type 2 diabetes share some similarities in the physiological basis of the disease, the course of the condition is very different meaning that these two conditions are likely to have a different psychological impact.

The findings of the telephone interviews in the present study are also largely consistent with another qualitative study that was conducted in the UK, but not based on psychological theory. In interviews with five non‐attenders in London, Campbell‐Richards et al reported that although participants placed high importance on attending appointments, they faced similar practical barriers to attendance such as transport, timing and frequency of appointments and competing demands for their time.^17^ Some participants also felt dissatisfied with the service they received consistent with the lack of value reported in the present study. However, the implicit finding underlying non‐attendance in the present study of a low perceived impact of diabetes was not reported in the Campbell‐Richards study. This unique finding of the present study suggests that psychological theory may be valuable in helping to understand why people with diabetes struggle to overcome some of the practical barriers to attending appointments.

In the present study gender, emotional representations, perceived behavioural control and attitude were significant predictors of the number of missed appointments in the regression. A recent systematic review of factors associated with attendance at diabetes appointments included no studies that assessed emotional representations, perceived behavioural control or attitudes.^18^ The review reported men were found to be more likely to miss appointments in some studies but that gender was not associated with attendance in most of the included studies.^18^ Gender was found to be a significant predictor of the number of missed diabetes appointments in the present study, but in the opposite direction to previous research with women being more likely to miss appointments than men. However, a study of non‐attendance at GP appointments found that women were more likely to miss appointments until the number of appointments made was controlled for, and then the opposite pattern was observed.^19^ We did not have access to the number of appointments made by participants in the present study, so it is possible that observed effect of female gender on attendance may have changed had we controlled for the number of appointments made. Previous research has assessed the ability of components of the SRM variables to predict attendance at diabetes appointments, but none has been identified using those of the TPB. Lawson et al^15^ found that perceptions of control predicted attendance at diabetes appointments. The present study found that emotional representation was the only one SRM variable that was significantly associated with an increased risk of missing diabetes appointments. The finding that people who perceive their condition to have a greater emotional impact are less likely to attend diabetes appointments warrants further investigation and appears to support recent calls for greater emotional support for people with diabetes.^20^ In addition, we found that TPB variables attitude and PBC were significantly associated with attendance with positive attitudes and lower PBC linked to a higher risk of missing appointments. The finding regarding PBC is consistent with the theory but the findings regarding attitudes are counter‐intuitive and cannot be explained. The findings of this study suggest that both the TPB and SRM may have utility in predicting attendance at diabetes appointments, but further research is required to explore this in a larger sample.

Limitations of this study relate primarily to the low response rate and corresponding small sample size. Because of the small sample size, the statistical analyses are likely to have lacked power to identify effects and the very low response rate may have resulted in a biased sample meaning that the findings may not be generalizable. The findings of this study therefore need to be interpreted with caution. The low response rate also meant that comparator interviews could not be conducted by the first author as planned but impressions formed by the first author during analysis were that interviews conducted by peer researcher provided useful data on the topic with the shared experience between peer researcher and interviewee seeming to assist in rapport building. It was noted though that peer researchers sometimes did not probe or follow up interesting statements made by participants which resulted in shorter, less in‐depth interviews than might have been achieved by a more experienced researcher. However, without comparator interviews these impressions cannot be confirmed and we cannot assess the effect of peer researchers on response rates.

People who do not engage with health care are known to be difficult to engage in research and often considered inaccessible.^15^ Much of the research investigating non‐attendance at health‐care appointments uses routinely collected health‐care data^21^, ^22^ or includes both attenders and non‐attenders^23^ thus overcoming difficulties with recruitment. However, research using routinely collected health‐care data is limited as it can only provide clinical and demographic information. Although the response rate was low and sample size small in the present study, the data collected provide an in‐depth exploration of psychological factors that influence attendance that was led by people who have diabetes themselves, and focused only on those who miss appointments. As such, it is hugely valuable in helping to understand people who do not attend appointments and the steps that might need to be taken to tackle non‐attendance.

Another limitation in the present study was that we asked participants to report the number of appointments they had missed as an absolute figure rather than a proportion. As non‐attendance was self‐reported, it was felt that it would be too difficult for people to calculate or recall the proportion of appointments that were missed. Although there will have been differences between participants in the number of appointments they were due to attend, it is unlikely that this difference would have been large as participants were all located in a single health board and would be receiving care based on the same clinical guidelines.

## 5 CONCLUSIONS

The findings of the present study highlight the importance of psychological variables in predicting non‐attendance at diabetes appointments and suggest that psychological variables, such as those from the TPB and SRM, could be of value in applied settings for identifying people with diabetes who are at risk of not engaging in health care relating to their diabetes. Whilst interventions to improve diabetes appointment attendance should address practical barriers to attending appointments, such as forgetting and the time and day of appointments, there may also be a need to take account of the underlying perceptions about diabetes, the emotional impact and perceived lack of value in attending appointments.

## CONFLICT OF INTEREST

The authors have no conflicts of interest to declare.

## Data Availability

The authors elect to not share data.

## References

[1] Nuti LA , Lawley M , Turkcan A , et al. No‐shows to primary care appointments: subsequent acute care utilization among diabetic patients. BMC Health Serv Res. 2012;12:304. 22953791 10.1186/1472-6963-12-304 PMC3470968

[2] Karter AJ , Parker MM , Moffet HH , et al. Missed appointments and poor glycemic control: an opportunity to identify high‐risk diabetic patients. Med Care. 2004;42:110‐115. 14734947 10.1097/01.mlr.0000109023.64650.73

[3] Paterson B , Charlton P , Richard S . Non‐attendance in chronic disease clinics: a matter of non‐compliance? J Nurs Healthc Chronic Illn. 2010;2:63‐74.

[4] Rhee MK , Slocum W , Ziemer DC , Culler SD , Cook CB , El‐Kebbi IM . Patient adherence improves glycemic control. Diabetes Educ. 2005;31:240‐250. 15797853 10.1177/0145721705274927

[5] Bonora E , Monami M , Bruno G , Zoppini G , Mannucci E . Attending Diabetes Clinics is associated with a lower all‐cause mortality. A meta‐analysis of observational studies performed in Italy. Nutr Metab Cardiovas Dis. 2018;28(5):431‐435. 10.1016/j.numecd.2018.02.009 29627120

[6] Lawson VL , Lyne PA , Bundy CE , Harvey J . The role of illness perceptions, coping and evaluation in care seeking among people with type 1 diabetes. Psychol Health. 2007;22:175‐191.

[7] Roberts S . Turning the corner: improving diabetes care. Department of Health. 2006 https://www.bipsolutions.com/docstore/pdf/13587.pdf. Accessed December 19, 2018.

[8] Domeq JP , Prutsky G , Elraiyah T , et al. Patient engagement in research: a systematic review. BMC Health Serv Res. 2014;14:89. 24568690 10.1186/1472-6963-14-89 PMC3938901

[9] Brett J , Staniszewska S , Mockford C , et al. Mapping the impact of patient and public involvement on health and social care research: a systematic review. Health Expect. 2012;17:637‐650. 22809132 10.1111/j.1369-7625.2012.00795.x PMC5060910

[10] Ajzen I . The theory of planned behaviour. Org Behav Hum Decis Process. 1991;50:179‐211.

[11] Leventhal H , Diefenbach M , Leventhal EA . Illness cognition: using common sense to understand treatment adherence and affect cognition interactions. Cognit Ther Res. 1992;16:143‐163.

[12] Broadbent E , Petrie KJ , Main J , Weinman J . The Brief Illness Perception Questionnaire (BIPQ). J Psychosom Res. 2006;60:631‐637. 16731240 10.1016/j.jpsychores.2005.10.020

[13] Francis J , Eccles MP , Johnston M , Walker AE , Grimshaw JM , Foy R , et al. Constructing questionnaires based on the theory of planned behaviour: a manual for health services researchers. Centre for Health Services Research. 2004 http://openaccess.city.ac.uk/1735/. Accessed December 4, 2018.

[14] Braun V , Clarke V . Using thematic analysis in psychology. Qual Res Psychol. 2006;3:77‐101.

[15] Lawson VL , Bundy C , Lyne PA , Harvey JN . Using the IPQ and PDMI to predict regular diabetes care‐seeking among patients with Type 1 diabetes. Br J Health Psychol. 2004;9:241‐252. 15125807 10.1348/135910704773891078

[16] Lawson VL , Lyne PA , Harvey JN , Bundy CE . Understanding why people with Type 1 diabetes don not attend for specialist advice: A qualitative analysis of the views of people with insulin‐dependent diabetes who do not attend diabetes clinic. J Health Psychol. 2005;10:409‐423. 15857871 10.1177/1359105305051426

[17] Campbell‐Richards D . Exploring diabetes non‐attendance: an inner London perspective. J Diabetes Nurs. 2016;20:73‐78.

[18] Lee RRS , Samsudin MI , Thirumoorthy T , Low LL , Kwan YH . Factors affecting follow-up non-attendance in patients with Type 2 diabetes mellitus and hypertension: a systematic review. Singapore Med J. 2019;60(5):216‐223. 31187148 10.11622/smedj.2019042 PMC6535449

[19] Ellis DA , McQueenie R , McConnachie Al , Wilson P , Williamson AE . Demographic and practice factors predicting repeated non-attendance in primary care: a national retrospective cohort analysis. Lancet Pub Health. 2017;2(12):e551‐e559. 29253440 10.1016/S2468-2667(17)30217-7 PMC5725414

[20] Diabetes UK . Future of Diabetes Report. Diabetes UK. 2017 https://www.diabetes.org.uk/resources-s3/201711/1111B%20The%20future%20of%20diabetes%20report_FINAL_.pdf. Accessed June 21, 2019.

[21] Williamson AE , Ellis DA , Wilson P , McQueenie R , McConnachie A . Understanding repeated non-attendance in health services: a pilot analysis of administrative data and full study protocol for a national retrospective cohort. BMJ Open. 2017 10.1136/bmjopen-2016-014120 PMC5319001 28196951

[22] Campbell K , Millard A , McCartney G , McCullough S . Who is least likely to attend? An analysis of outpatient appointment ‘did not attend’ (DNA) data in Scotland. NHS Health Scotland. 2015 http://www.healthscotland.scot/media/1131/5317_dna-analysis_national.pdf. Accessed December 21, 2018.

[23] Masuda Y , Kubo A , Kokaze A , et al. Personal features and dropout from diabetic care. Environ Health Prev Med. 2006;11(3:115‐119. 21432385 10.1265/ehpm.11.115 PMC2723222

